# Orientation of tyrosine side chain in neurotoxic Aβ differs in two different secondary structures of the peptide

**DOI:** 10.1098/rsos.160112

**Published:** 2016-10-05

**Authors:** Swagata Das, Supriya Das, Anupam Roy, Uttam Pal, Nakul C. Maiti

**Affiliations:** Structural Biology and Bioinformatics Division, Indian Institute of Chemical Biology, Council of Scientific and Industrial Research, 4, Raja S.c. Mullick Road, Kolkata 700032, India

**Keywords:** Aβ peptide, tyrosine fluorescence, conformation, rotamer

## Abstract

Amyloid β (Aβ) peptide is present as a major component in amyloid plaque that is one of the hallmarks of Alzheimer's disease. The peptide contains a single tyrosine residue and Aβ has a major implication in the pathology of the disease progression. Current investigation revealed that the tyrosine side chain attained two different critical stereo orientations in two dissimilar conformational states of the peptide. The extended α-helical structure of the peptide observed in an apolar solvent or methanol/water mixture became disordered in aqueous medium and the radius of gyration decreased. In aqueous medium, the torsional angle around C_α_–C_β_ of tyrosine group became −60°. However, in its α-helical conformation in an apolar system, the measured angle was 180° and this rotameric state may be reasoned behind stronger tyrosine fluorescence compared with the disordered state of the peptide. Molecular dynamics simulation analyses and spectroscopic studies have helped us to understand the major structural changes in the secondary structure of the peptide in the two conformational states. A conformational clustering indicated that the compact state is more stable with tyrosine residue attaining the torsion angle value of −60°, whereas the native state (in HFIP/water mixture) is prevalent at a torsion angle value of −180°. High solvent accessibility has possibly stabilized the particular rotameric state (−60°) of the tyrosine residue and could be the reason behind decrease in fluorescence of the sole tyrosine residue in an aqueous buffer solution (pH 7.4) compared with its fluorescence in the α-helical structure in the micellar environment.

## 1. Introduction

Alzheimer's disease (AD) is a progressive mental depreciation leading to damage of the brain cells and mental functions, often leading to memory loss. The disease is often linked to the deposition of amyloid proteins (Aβ) in the brain, which cause plaque formation and eventual loss of brain functions [1–5]. Amyloid precursor protein, a large integral membrane protein [6], is sequentially cleaved by two membrane-bound endoproteases, β- and γ-secretases resulting in C-terminal heterogeneity of the resulting peptide population. Hence, different Aβ species exist, but those ending at position 40 (Aβ40) are the most abundant (approx. 80–90%), followed by 42 (Aβ42, approx. 5–10%). Aβ42 is more hydrophobic and fibrillogenic, and during AD, Aβ42 fibre is one of the major species deposited in the brain [7,8]. This fibrillar structure on large surface of the cell membrane is believed to be one of the major causes of brain cell damage and death. Recent studies and hypothesis suggested that not only the fibrillar aggregates but oligomeric and protofibrillar structures, intermediates in the process of fibril formation, may also be neurotoxic in nature [9–16].

The amyloidogenic peptide Aβ42 lacks a particular structure in a physiological condition, and it can also transform into insoluble aggregates of different morphology with a major β-sheet content in test tube experiments [7,17–20]. Thus, the observation that the peptide easily can form amyloid aggregates *in vitro* and *in vivo* and its link to amyloidogenesis is significantly established; however, the mechanism by which this water-soluble peptide easily transforms into β-sheet-rich aggregates and other intermediates is not well known. The general hypothesis is that Aβ peptide with largely disordered conformation first coalesces to form small soluble oligomers [13,21,22], which subsequently reorganize and assemble into long and insoluble beta fibrils which are rich in β-sheet structure [23,24]. Thus, the peptide conformation plays a crucial role in the aggregation processes.

**Scheme 1.**
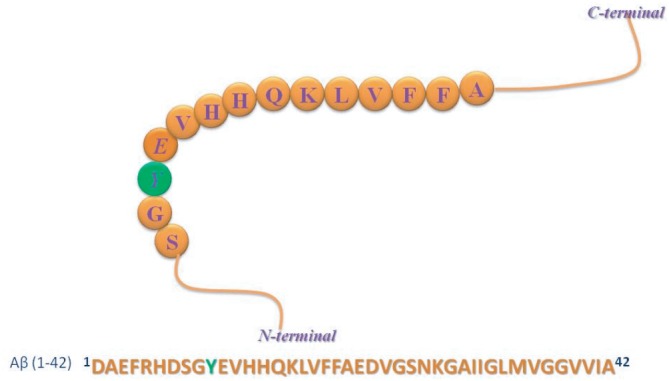
Amino acid sequence of amyloid beta peptide, Aβ42. Tyrosine residue at position 10 is marked green.

Changes in protein/peptide conformation and the interaction of proteins with other molecules are often investigated using fluorescence of tryptophan and/or tyrosine residues present in proteins and peptides [25–28]. The fluorescence properties of tyrosine residue in the Aβ peptides can thus effectively provide structural details of the peptides and could be very useful in mechanistic investigation of the aggregation process and its interaction with other molecules [29,30]. In addition, the position of the tyrosine residue in the Aβ peptide is in an important domain, presiding in the very close proximity of three histidine residues (HDSGYEVHH) (scheme 1); those are directly involved in metal coordination [31,32]. Close proximity of the tyrosine residue to the neighbouring histidines makes it sensitive for metal-induced chemical changes that may have serious implication in amyloid formation [1,7,20]. Maiti *et al*. [33] used the tyrosine fluorescence to realize the coordination pattern of the peptide with Cu(II). We showed here that the side chain of Tyr10 attained a critical stereo orientation and the torsional angle (*χ*) around C_α_–C_β_ became −60° in the disordered state. However, in an α-helical conformation of the peptide, the tyrosyl orientation was altered and the angle became 180°. Also we observed a good amount of changes in secondary structural element among the residues neighbouring to the tyrosine residue.

## 2. Material and methods

### 2.1. Sample preparation

Aβ peptides were purchased as lyophilized solids from American Peptide Company, Inc., Sunnyvale, CA, USA. All other chemicals were purchased either from Fisher Scientific, Sigma Aldrich Chemicals or Merck. A typical Aβ42 peptide solution was prepared by adding 200 µl of 20 mM NaOH solution to 0.1 mg of the peptide and shaking for few minutes. The highly alkaline buffer ensures that the peptide is maintained in its monomeric condition [34,35]. The resultant solution was centrifuged for 20 min at 15 000*g* rcf to remove any insoluble particles. The experiments were performed with the supernatant in order to avoid any interference of the insoluble particles. The concentration was determined from the UV absorption of the tyrosine (ε_275_ = 1410 M^−1^ cm^−1^) residue at pH 7.4 [33]. Concentrated Aβ solution was further diluted in aqueous buffer or in 0.5% sodium dodecyl sulfate (SDS) solution.

### 2.2. Equipment and measurements

#### 2.2.1. Circular dichroism

Circular dichroism (CD) measurement was conducted on a JASCO 710 spectropolarimeter at room temperature using a 0.1 mm path length quartz cell with a 300 µl capacity. The peptide (approx. 20 µM) solutions in different solvents were prepared and immediately CD spectrum was recorded. Each representative spectrum was an average of four to eight scans.

#### 2.2.2. Fluorescence

The steady-state fluorescence measurement of Aβ42 solutions (approx. 20 µM) at room temperature was carried out using an Agilent Vary Eclipse spectrofluorimeter. Experimental conditions were *λ*_ex_ =  280 nm, with both entrance and exit slit widths set at 10 nm. All the spectra were corrected by subtracting the background signals from the solvent. The relative quantum yield was determined from the following equation:

2.1 $$\phi_{\mathrm{sample}}=\frac{F_{\mathrm{sample}}}{F_{\mathrm{tyrosine}}}\times \frac{A_{\mathrm{tyrosine}}}{A_{\mathrm{sample}}}\times \phi_{\mathrm{tyrosine}},$$

where *F*_sample_ and *F*_tyrosine_ are the measured fluorescence intensities (areas under the fluorescence peaks) of the sample (Aβ42) and the reference (tyrosine in aqueous buffer), respectively. *A*_sample_ and *A*_tyrosine_ are the absorbance values at the same excitation wavelength (280 nm) of the sample and the reference solutions, respectively. Here *ϕ*_sample_ and *ϕ*_tyrosine_ are the quantum yields of the sample and tyrosine solutions, respectively. The measured quantum yield was relative to tyrosine and the value for the tyrosine in aqueous buffer was assumed 1.

### 2.3. Molecular dynamics

The molecular dynamics (MD) simulation was carried out using Desmond as implemented in Schrödinger Maestro 2015–4 package with 50 ns (nanoseconds) simulation time. The MD simulation was carried out starting with the Aβ42 peptide structure obtained from NMR structure (Protein Data Bank (PDB) ID: 1IYT) [36]. An ensemble of 20 conformations was given in the PDB structure mentioned above. Three trajectories were obtained with different initial conformations of the peptide. Maestro protein preparation wizard was used to fix erroneous atomic representations in structure. The system for MD simulations was built by embedding the peptide with an aqueous layer using simple point charge (SPC) model to describe the water molecules; box size was 60 × 60 × 60 Å^3^. Six chloride ions were added to charge neutralize the whole system. The peptide was placed at the centre of the box and the distance from the boundary was at least 10 Å. Optimized potentials for liquid simulations (OPLS) 2005 force field was used for the simulation [37]. Five step relaxation protocol was used starting with Brownian dynamics for 100 ps with restraints on solute heavy atoms at NVT (with *T* = 10 K) followed by 12 ps of dynamics with restraints at NVT (*T* = 10 K) and then at NPT (*T* = 10 K) using Berendsen method. Then the temperature was raised to 300 K for 12 ps followed by 24 ps relaxation step without restraints on the solute heavy atoms. The production MD was run at NPT with *T* = 300 K for 50 000 ps. MD results were analysed using the simulation event analyser embedded in Desmond/Maestro. Metadynamics simulation was also performed using Desmond. Protein preparation steps and the relaxation protocol used for the metadynamics simulation were similar to those mentioned above. Torsional angle was perturbed for 360° scan, with step size 5°.

## 3. Results and discussions

**Figure 1.**
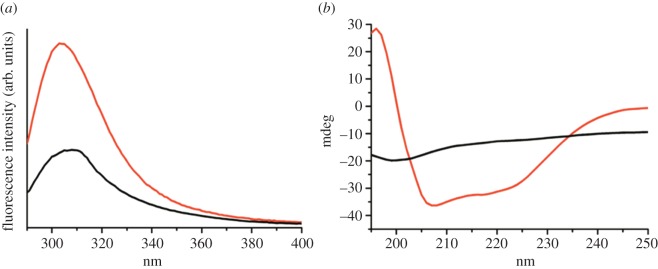
Fluorescence (*a*) and CD spectra (*b*) of Aβ peptide. In panel (*a*), fluorescence spectra of 20 µM Aβ42 in 10 mM sodium phosphate buffer at pH 7.4 (black), and in 0.5% SDS solution in water (red) are shown. Spectra were recorded at room temperature, *λ*_ex_ = 280 nm. The plots show fluorescence peak at 304 nm in SDS micelle as well as buffer solution (24°C). CD spectra of the respective solutions at room temperature are shown in panel (*b*), black in the buffer and red in SDS micelle.

We observed different degrees of fluorescence from the tyrosine residue in two different secondary structures of the peptide. With an α-helical conformation state, the peptide showed stronger fluorescence compared with its intrinsic disordered conformation. Figure 1*a* displays the solvent-corrected fluorescence spectra of the peptide in SDS micelle and in aqueous buffer. Upon excitation at 280 nm, the peptide in aqueous buffer displayed fluorescence with a band maximum at approximately 304 nm. This profile was typical of commonly observed tyrosine fluorescence in water. However, the peptide fluorescence was slightly broader than free l-tyrosine. The relative fluorescence quantum yield (Φ) with respect to l-tyrosine at pH 7.4 was approximately 0.2. The peptide remained disordered in this pH and showed a negative CD band at approximately 198 nm (figure 1*b*). However, in SDS micelle, the fluorescence band of Aβ42 appeared at a similar position and the fluorescence quantum yield (Φ ∼ 0.5) was higher than that in aqueous solution with disordered conformation.

**Figure 2.**
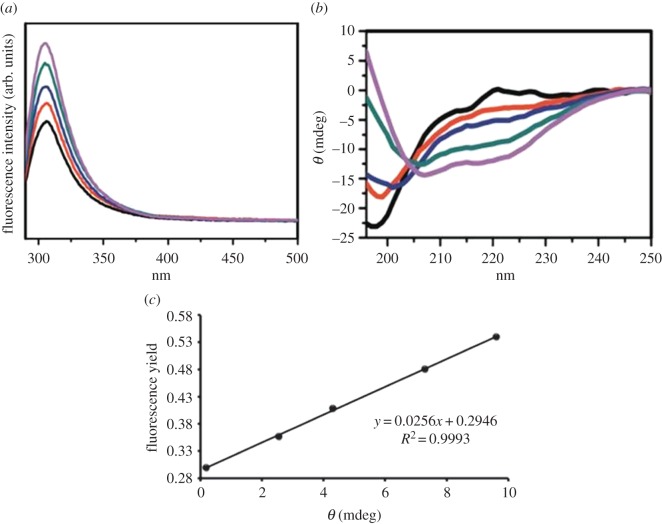
(*a*) Fluorescence spectra of Aβ42 in different concentration of methanol in water. Black, 10% (v/v) methanol; red, 20% (v/v) methanol; blue, 30% (v/v) methanol; green, 40% (v/v) methanol and purple, 50% (v/v) methanol. Fluorescence measurement conditions were similar to figure 1. (*b*) CD spectra of Aβ in respective solutions and different colours indicating the presence of different percentage of methanol as panel (*a*). (*c*) The plot of relative quantum yield (*Φ*) versus relative ellipticities at 222 nm (Φ_222_). The data points tilted to a straight line.

To examine the role of intricate α-helical conformation to the fluorescence of the tyrosine residue, we measured the fluorescence quantum yield (Φ) with varying the α-helicity by adding methanol to the peptide solution. Figure 2*a* shows the change in the fluorescence of Aβ at various concentrations (%) of methanol. The fluorescence intensity at approximately 304 nm enhanced as the concentration of methanol increased in the peptide solution. Conformation of the peptide in the presence of methanol was measured by CD spectroscopy (figure 2*b*). In the absence of methanol, the negative band that appeared at approximately 198 nm defined the flexible and disordered structure of the peptide. However, the peptide became more α-helical as the concentration of methanol was increased; two negative bands at 208 and 222 nm started to appear in the CD spectrum. This confirmed that the methanol/water mixture induced formation of α-helical structure in the peptide, and these structural changes influenced the fluorescence intensity of the peptide without major alteration of the florescence band position (figure 2*a*). Figure 2*c* shows a plot of fluorescence quantum yield (Φ) versus observed ellipticity (Θ) of the peptide at 222 nm and it was fitted to a straight line. Thus, the relative fluorescence quantum yield was directly correlated with the α-helical content of the peptide.

**Figure 3.**
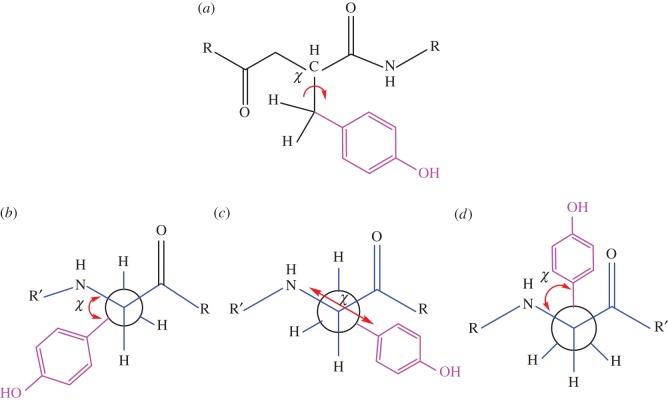
Schematic diagram (*a*) depicting C_α_−C_β_ bond rotation of tyrosine residue in the peptide along with different rotameric conformations of tyrosine shown in Newman projection based on the dihedral angle (*χ*, chi); gauche(−), *χ* = −60° (*b*), trans, *χ* = 180° (*c*) and gauche (+), *χ* = +60°(*d*).

The solvent-induced α-helix conformation possibly influenced the residue to favourably attain a particular rotameric position that can produce stronger fluorescence due to specific orientation of the tyrosine residue that produced the fluorescence. Rolinski *et al.* [38,39] studied the tyrosine fluorescence in Aβ and used a three-rotamer model (figure 3) to explain the main fluorescence behaviour of the peptide monomer in several solution conditions. Tyr10 stable rotamers, as mentioned earlier, could be defined with three different dihedral angles (*γ*) of 180°, 60° and −60° (figure 3). The tyrosyl moiety in each rotameric orientation/state experiences a different microenvironment; it remained at different distances from the adjacent peptide linkages, some of which may contribute to the fluorescence quenching. Fluorescence quenching via excited state charge transfer often depends on the rotameric structure of the fluorophore moiety [40–47]. Three different fluorescence lifetimes were suggested for each of the rotameric states of the tyrosyl group in aqueous buffer at low (micromolar range) peptide concentration [39]. The rotameric component that produces higher fluorescence (lifetime) might be significantly less in low concentration of the peptide solution [39]. We observed that the microenvironment created surrounding the peptide, both in SDS micelle and water/alcohol mixture, enhanced the fluorescence of the peptide. This indicated that the rotameric state that produces higher fluorescence was populated when the peptide attained α-helical conformation. In aqueous buffer, the peptide remained largely disordered and the tyrosine residue may attain a rotameric state that produces less fluorescence and is quite different to that in the α-helical structure.

**Figure 4.**
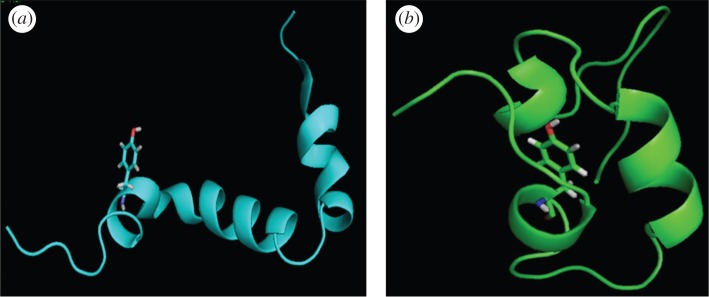
Snapshots of Aβ42 obtained from MD simulations of the peptide in water. At the initial conformation, the dihedral angle (*χ*) of tyrosine was 180° and after simulation of 49 ns the peptide oriented itself to the conformation where quenching by the carbonyl group at tyrosine was least, i.e. *χ* = −60°. The figures were generated and all the measurements were performed using Schrödinger Maestro and PyMOL. Tyr10 was protruded to have a better view.

To realize the rotameric state of the tyrosyl group and the positioning of surrounding residues which could influence the overall fluorescence, MD analysis was carried out. The simulation was done with the Aβ42 peptide structure obtained from PDB entry 1IYT [36]. The peptide in its native disordered state (initial elongated conformation) is shown in figure 4*a*, where the tyrosine residue is in the −180° rotational state. Figure 4*b* shows the compact structure which is more favoured as obtained from MD simulation. In this compact structure, the tyrosine residue remains in −60° rotational state.

**Figure 5.**
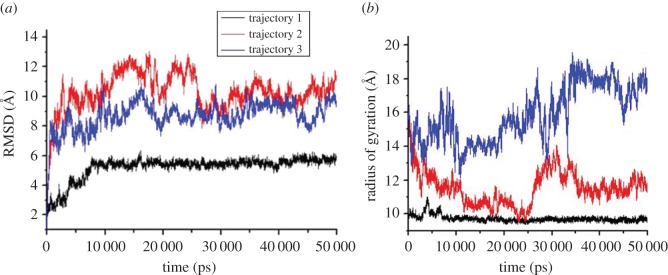
The changes in RMSD (*a*) and radius of gyration (*b*) for the three trajectories are shown.

Figure 5*a* shows the changes in all atom RMSD values of whole peptide chain with respect to MD timescale. Three trajectories from three independent simulations are shown. Each simulation was started with different models from the PDB ID 1IYT. Figure 5*a* suggests that the peptide reached a stable conformation after a certain period of time. Figure 5*b* shows the radius of gyration (Rg) plot from the three different MD simulations. Rg is an indicator of compactness of the structure. Decrease in the Rg value indicates more compact conformation. In trajectory 1, formation of the stable compact structure was observed, whereas in trajectories 2 and 3, some fluctuations in the Rg value were observed over the simulation timescale.

**Figure 6.**
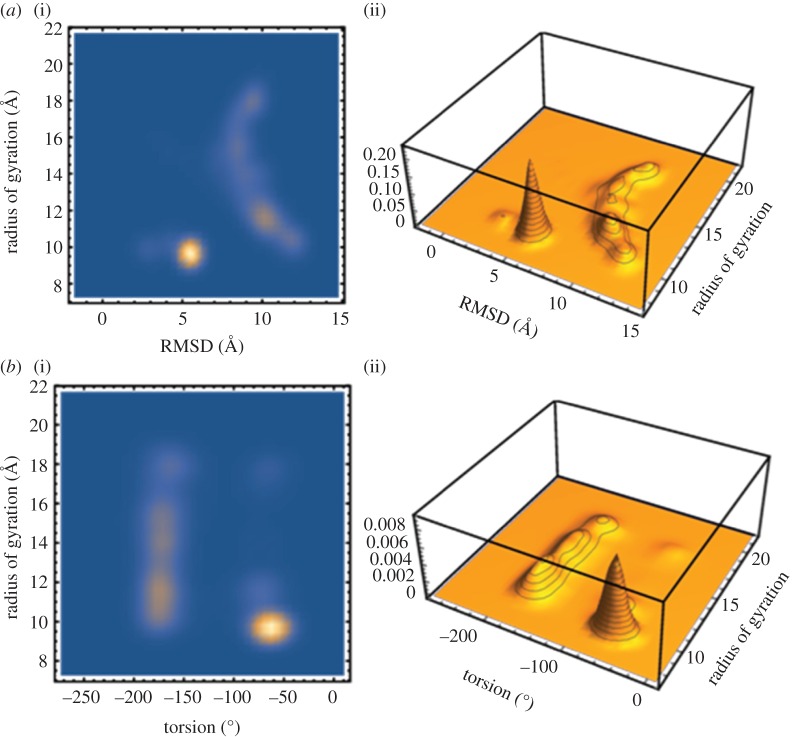
*a*(i,ii) The conformational clustering based on the radius of gyration with RMSD values obtained from the sets of trajectory, with contour plot of radius of gyration with RMSD (i) and three-dimensional histogram plot (ii). *b*(i,ii) The conformational clustering based on correlation between radius of gyration and dihedral/torsion angle obtained from the sets of trajectory, with contour plot of radius of gyration with dihedral angle (i) and three-dimensional histogram plot (ii).

We further performed a conformation clustering correlating the Rg and RMSD values to better understand the distribution of peptide conformation and to find out the most populated state of the Aβ peptide. A contour plot showing the correlation of RMSD and Rg value is shown in figure 6*a*. A compact structure with an Rg value of 9.5 Å and a standard deviation of 5.5 Å from the initial conformation (PDB structure 1IYT) was observed more frequently in the simulation timescale. We have also performed a conformational clustering based on the torsion angle versus radius of gyration plot which is shown in figure 6*b*. The figure shows that the compact state, which is statistically more prominent, has a stable value of tyrosine at −60°, whereas, the native structure is more prone to attain −180° conformation.

**Figure 7.**
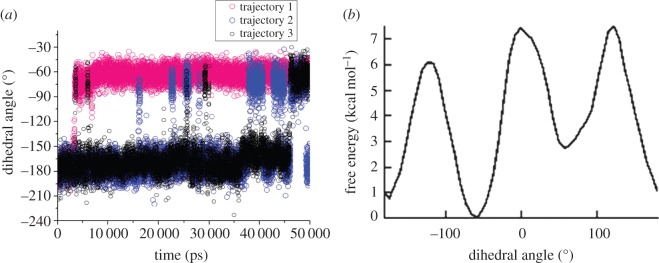
(*a*) The dihedral angle around the C_α_–C_β_ bond of tyrosyl throughout the three trajectories (blue, black and red) of MD simulation timescale has been shown. The dihedral angle fluctuates between −180° and −60°. However, two states are highly populated, one at −180° and the other at −60°. (*b*) Energy diagram for the rotameric conversion of tyrosine sidechain. Molecular conformation with tyrosine dihedral angle −60° is energetically more stable as obtained from metadynamics study.

We have done metadynamics simulation to gain insights into the energetics of the rotameric conversion of tyrosine residue. The energy diagram for the rotameric conversion is shown in figure 7. The metadynamics analysis clearly shows that the −60° rotameric state is the energetically most favourable state. Here the −180° rotameric state is also energetically favourable and the energy barrier between these two states is 6 kcal mol^–1^.

**Figure 8.**
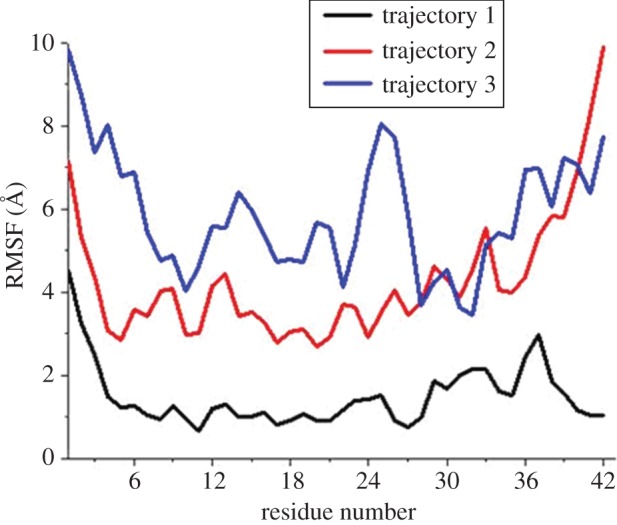
The changes in the RMSF for the three trajectories is shown.

**Figure 9.**
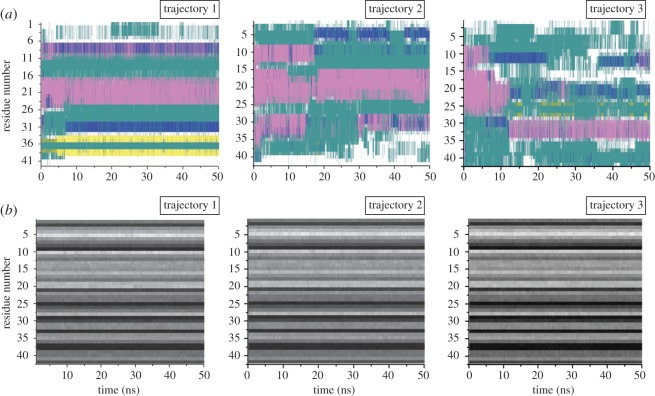
Obtained SSE and SASA of the peptide throughout the three MD simulation trajectories. (*a*) SSE like α-helices and β-strands are monitored throughout the simulation trajectory. The panel reports the SSE composition from helices and strands over frames obtained from the trajectory for each amino acid residue. The different colour code indicates different SSE present. Region 18–24 has almost retained the helical structure throughout the trajectory whereas the Tyr10 has emerged with the region having several SSE changes consisting of helices (pink) and turns (green). Blue indicates the 3_10_ helix, yellow indicates the β-strands and white indicates coil conformation. (*b*) The SASA of the peptide has been shown throughout the simulation trajectory. Tyr10 remains exposed to the solvent throughout the simulation, making it accessible for H-bonding with the water molecules of the microenvironment.

Figure 8 gives the root mean square fluctuation (RMSF) plot of the amino acid residues of the peptide with respect to the entire MD simulation. We observed that Tyr10 residue did not undergo much change in conformational fluctuations, whereas for other residues such as regions 1–4, 21–25, 28–38 the RMSFs were more. Furthermore, the secondary structure elements (SSE) like α-helices and β-strands were monitored throughout the simulation trajectory and we observed a significant amount of structural adjustment. Figure 9*a* gives the SSE composition (helices and strands) over frames obtained from the trajectory for each amino acid residue. It was observed that the region 18–24 has almost retained the helical conformation throughout the trajectory, whereas concerned Tyr10 has emerged with a significant changes in SSE of the neighbouring residues.

The helical structure consisting of two regions Ser8-Gly25 and Lys28-Val40 with a turn in between was unfolded to the conformation composed with loop region at Asp7-Tyr10 and helical structure at region Val18-Val24 and Gly28-Ile32. The torsional angle (*χ*) of tyrosine was 180° in the starting conformation and during simulation, the peptide oriented itself to the conformation in which *χ* became −60° (figure 3). This indicates that Tyr10 might undergo rotation and change its inclination from Gly9 towards Glu11 over the MD simulation time.

The solvent accessible surface area (SASA) indicated that Tyr10 remained exposed to the solvent throughout the simulation (figure 9*b*). So, it could be possible that it forms an H-bond with the water molecule accessible in the aqueous microenvironment. The rotameric state (*χ* = −60°) may be a highly populated species in aqueous buffer and it may be involved in stronger interaction with the Glu11/Asp7, or in H-bond formation with water, if not in the ground state in the first excited state. This interaction depends on the *p*Ka of the tyrosine hydroxyl group [48,49]. The hydroxyl group of l-tyrosine ionizes in the ground state with *p*Ka ∼ 10.3 [50] in aqueous buffer; however, it is more acidic in the first excited singlet state with an estimated *p*Ka of 4–5.2 [51]. The excited state of the tyrosyl group in disordered conformation was likely to decrease and may be closer to the excited state *p*Ka of l-tyrosine. In the excited state, the *p*Ka of the tyrosine OH reduced in disordered conformation and enhanced the possibility of formation of hydrogen bonding and ionic interaction with the surrounding group. Buried tyrosine residues in proteins show higher *p*Ka [51,52]. In α-helical conformation with a rotameric state of *χ* = 180° the tyrosine group *p*Ka may be higher and produce stronger fluorescence. Thus, MD analysis and fluorescence investigation suggested that the tyrosine residue remained separate in two different stereo orientations in two different conformation states of the peptides, and interacted differently with surrounding residues and solvent molecules.

Fluorescence is a molecular property and its geometry and electronic configuration that include rotameric structures, both in the ground state and excited state, have significant roles in the fluorescence property of a molecule. In proteins and peptides, intrinsic chromospheres like tryptophan and tyrosine residues often produce fluorescence. The time-resolved fluorescence decay analysis often found that fluorescence decay fit to multi-component lifetime components and with different amplitudes. Some experimental and theoretical investigation with tryptophan- and tyrosine-containing proteins and peptides suggested the existence of rotameric states as the source of different fluorescence components [40–47,53–55]. Different degrees of fluorescence quenching are possible as the excited state charge transfer depends on the rotameric structure of the aromatic component, an indole ring in the case of tryptophan residue and phenole ring for tyrosine residue. It also depends on the charge acceptor groups. The torsional angle (*χ*) of tyrosyl was 180° in the starting conformation (helical structure) and disordered conformation (as observed in molecular dynamic analysis) peptide oriented itself to the conformation in which *χ* became −60° (figure 3). These changes in rotameric state of the tyrosyl group may alter the charge transfer mechanism and produce different degrees of fluorescence [44]. Also, it was mentioned in an earlier report that the tyrosine fluorescence peak position is less sensitive to the polarity of the solvent, though tryptophan emission is very sensitive to local environments [53–55]. Upon increasing the methanol concentration the polarity of the solution changed; however, emission position was less affected and it may be due to intrinsic nature of the tyrosyl group itself [53–55]. The tyrosine residue in Aβ produced fluorescence that depended on the peptide conformation. We also found that the peptide with α-helical secondary structure produced enhanced fluorescence intensity and that may be due to specific rotameric structure of the tyrosyl side chain and different kind of charge transfer interaction.

## Data accessibility

This work did not have any supplementary experimental data.
